# Interpersonal emotion regulation and symptom dimensions of psychosis proneness in young adults

**DOI:** 10.1038/s41537-024-00520-x

**Published:** 2024-11-01

**Authors:** Marcel Riehle, Hannah Allmandinger, Luise Pruessner

**Affiliations:** 1 University of Hamburg, Institute for Psychology, Hamburg, Germany; 2 Witten/Herdecke University, Department of Psychology and Psychotherapy, Witten, Germany; 3 Heidelberg University, Department of Psychology, Heidelberg, Germany

**Keywords:** Schizophrenia, Psychosis, Human behaviour

## Abstract

This study investigated the relative associations of psychosis proneness symptom domains with habitual interpersonal emotion regulation (IER) use in a sample of young adults (*n* = 420, age 18–29). Multiple regression models showed that attenuated negative symptoms were related to using *less*, while attenuated positive symptoms and depression were related to using *more* IER. These findings suggest symptom-specific IER patterns across different symptom dimensions of psychosis proneness.

Roughly two-thirds of emotion regulation (ER) episodes are estimated to include regulation through social interaction^1^, i.e., *interpersonal emotion regulation*^2^ (IER). Despite this preponderance of IER, the efforts that have led to the identification of ER alterations in psychotic disorders^3^ and their risk states^4^ have revolved mainly around *intra*personal ER strategies. However, disruptions in IER could be a mechanistic link explaining how the social disconnect associated with psychosis-risk states^5^ fosters transition to psychosis^6^.

Recent studies that have started to systematically investigate the link between IER and psychotic symptoms focused on schizotypal traits^7,8^ and psychosis proneness^9^. They identified symptom domain-specific associations with altered IER use on a habitual level^7,9^ and when assessed in an everyday setting^9^. Negative schizotypal traits and attenuated negative symptoms were associated with seeking the company of others for comfort, for advice on how to deal with a distressing situation, and to sustain or enhance positive affect *less*^7,9^. Positive schizotypal traits and attenuated positive symptoms, in contrast, were associated with *increased* use of IER to deal with distressing emotions^7,9^.

The present report aimed to test for the relative associations of three symptom domains of psychosis proneness covered by the Community Assessment of Psychic Experiences^10^ (CAPE; i.e., attenuated positive and negative symptoms and depression), with habitual IER use in young adults (age 18–29). This study expands on prior research by addressing gaps in earlier studies^7–9^. Specifically, it combines a focus on young adulthood, a critical period for the onset of psychotic disorders^11^, with a broader assessment of multiple IER strategies and a wider range of symptom severity levels. While previous studies^7–9^ have addressed some of these elements, none has simultaneously integrated all these aspects within a large sample.

The present study was conducted online between October 2021 and July 2022 and was approved by the local ethics committee of the University of Hamburg (2021_394_Bach_Dalmis_Riehle). The sample consisted of 420 young adults (age: *M* = 22.9, *SD* = 3.1; gender: 79.8% female, 19.0% male, 1.2% diverse; minority status: 20.7%; additional sample characteristics are reported in the supplementary material) who took the survey as a screening for a subsequent 7-day diary study that involved about one-quarter of the present sample and which has been reported elsewhere^9^.

The online survey was implemented in Limesurvey (Limesurvey GmbH, Germany) and included, in this order: informed consent, demographics, the CAPE, the Interpersonal Emotion Regulation Questionnaire^12^ (IERQ), and the Emotion Regulation Questionnaire^13^ (ERQ). The IERQ assesses four habitual IER strategies^12,14^: enhancement of positive affect (IERQ-EP, Cronbach’s *α* in this sample = 0.83), social modeling (IERQ-SM, α = 0.83), soothing (IERQ-SO, α = 0.86), and perspective-taking (IERQ-PT, α = 0.81). The ERQ assesses two habitual intrapersonal ER strategies, expressive suppression (ERQ-ES, α = 0.73) and cognitive reappraisal (ERQ-CR, α = 0.84).

Participants were recruited via various online platforms, including platforms for course credit administration at the University of Hamburg. Additionally, flyers were sent to early psychosis detection centers throughout Germany and Austria. The only inclusion criterion was an age between 18 and 29 with the aim of including a symptomatically diverse sample. As shown in Fig. 1, the sample showed a diverse pattern of attenuated psychotic symptoms. CAPE negative symptoms (*M* = 1.06, *SD* = 0.47, range = [0.00, 2.71], *α* = .86) and depression (*M* = 1.11, *SD* = 0.51, range = [0.13, 3.00], *α* = 0.84) were approximately normally distributed in the sample. CAPE positive symptoms (*M* = 0.48, *SD* = 0.31, range = [0.00, 1.90], *α* = 0.84) showed typical skew^15^.

**Fig. 1:**
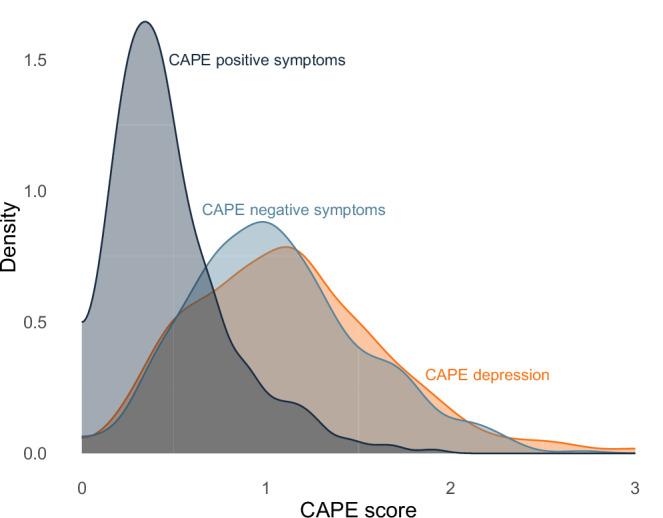
Density plot of the three symptom dimensions of the CAPE^10^ showing the distribution of scores in the sample (*n* = 420) pooled from 30 multiply imputed data sets.

Participants were included in the analysis if they completed the demographic questionnaire. Missing data (7.4% of cases) and responses from participants with identified unreliable response patterns (2.4% of cases) were multiply imputed using the *mice* package in R (version 4.2.3) (see supplement for details). Multiple regression analyses were conducted to test associations between CAPE symptom domains and IERQ and ERQ subscales, controlling for gender, minority status, and age. Analyzing the three symptom dimensions concurrently allowed control for general, nonspecific psychopathology and accounted for overlap among the symptom dimensions. The false discovery rate was controlled at *p* < .050 using the Benjamini-Hochberg correction.

Regression results are shown in Table 1 (bivariate correlations and full regression models are reported in the online supplement). CAPE negative symptoms predicted a *diminished* use of seeking out others to sustain or enhance positive affect (IERQ-EP), for comfort (IERQ-SO), and for advice on how to deal with a distressing situation (IERQ-SM). CAPE positive symptoms predicted an *increased* use of seeking out others for different perspectives on a distressing situation (IERQ-PT). CAPE depression predicted an *increased* use of seeking out others to sustain or enhance positive affect (IERQ-EP) and for comfort (IERQ-SO) and a *decreased* use of seeking out others for different perspectives on a distressing situation (IERQ-PT). Regarding intrapersonal ER, CAPE negative symptoms predicted an *increased* use of expressive suppression (ERQ-ES) and CAPE depression a *decreased* use of cognitive reappraisal (ERQ-CR).

**Table 1:** Results of the multiple linear regression analyses testing for relative associations of psychosis proneness symptom domains with habitual inter- and intrapersonal emotion regulation.

DV: Interpersonal ER	*R²*	β	*p*	DV: Intrapersonal ER	*R²*	β	*p*
IERQ-EP	0.10			ERQ-ES	0.15		
CAPE positive		0.06	0.233	CAPE positive		−0.03	0.585
CAPE negative		−**0.40**	**<0.001**	CAPE negative		**0.39**	<**0.001**
CAPE depression		**0.19**	**0.005**	CAPE depression		−0.01	0.911
IERQ -SM	0.05			ERQ-CR	0.07		
CAPE positive		0.11	0.044	CAPE positive		0.08	0.164
CAPE negative		−**0.19**	**0.009**	CAPE negative		−0.08	0.279
CAPE depression		0.02	0.757	CAPE depression		**−0.21**	**0.004**
IERQ-SO	0.07						
CAPE positive		0.04	0.472				
CAPE negative		−**0.32**	**<0**.**001**				
CAPE depression		**0.23**	**0**.**001**				
IERQ-PT	0.13						
CAPE positive		**0.34**	**<0.001**				
CAPE negative		−0.14	0.050				
CAPE depression		−**0.16**	**0.021**				

*Note: N* = 420. *DV* dependent variable, *ER* emotion regulation, *CAPE* Community Assessment of Psychic Experiences^10^; *IERQ-EP, -SM, -SO, -PT* Interpersonal Emotion Regulation Questionnaire^12,14^, subscales: enhancement of positive affect, social modeling, soothing, perspective taking, *ERQ-ES, -CR* Emotion Regulation Questionnaire^13^, subscales: expressive suppression, cognitive reappraisal. All analyses additionally controlled for age, gender, and minority status (full model results reported in the supplement). All VIFs ≤ 2.21; not suggesting multicollinearity. The presented standardized estimates were pooled according to Rubin’s rules from 30 multiply imputed data sets. Significant estimates after Benjamini-Hochberg correction are printed in bold font.

These findings corroborate previous research^7,9^ by showing symptom domain-specific relative associations of psychosis proneness and IER alterations in young adults. Particularly interesting is that attenuated negative symptoms were related to diminished, but attenuated positive symptoms and (in part) depression were linked to increased use of IER strategies when positive, negative, and depressive symptoms were tested as concurrent predictors in a single regression model. Fewer opportunities to sustain or enhance positive affect^9,16^ and low expectations of others being helpful resources^17–20^ are likely explanations of the low IER use associated with attenuated negative symptoms. In contrast, the associations of attenuated positive symptoms and depression with increased IER use are likely explained by heightened negative affect and reassurance-seeking associated with these symptom domains^9^.

Several limitations of this work need consideration. First, the sample was highly educated and predominantly female, limiting the generalizability to other demographics, including samples of people with clinical high risk for psychosis, which typically include a larger proportion of male participants^21^. Second, the range of attenuated positive symptoms was restricted, limiting the generalizability to more severe positive symptom levels. This restricted range could also signify a sampling bias, and we cannot rule out that our findings for attenuated positive symptoms are explained by a protective effect of IER against symptom worsening. Third, the cross-sectional design prevents conclusions about the causal direction of the associations reported here. Fourth, subtypes of the symptom dimensions tested here could have differential effects on IER, and future research should investigate this possibility. For example, in our diary study^9^, we found effects for paranoid ideation that were similar to those for positive symptoms in the present study. Finally, the IERQ assesses self-focused IER strategies and presumes explicit regulation goals^2,12^, not accounting for potential other-focused or implicit IER strategies. The large sample of young adults covering a wide range of the continuum of attenuated negative symptoms and depression is a particular strength of this study.

In conclusion, this study adds to the emerging evidence suggesting that a diminished use of IER is a relevant aspect of the social disconnect specifically associated with attenuated negative symptoms in young adults. The specificity of the role of negative symptoms for this disconnect is underscored by findings showing that young adults who experience attenuated positive symptoms, such as suspiciousness, turn to others *more* in times of distress.

## Supplementary information

Supplementary material

## Data Availability

All data and analysis code needed to reproduce the analyses and findings of this study are publicly available for non-commercial scientific use at psycharchives.org (data: 10.23668/psycharchives.15527; code: 10.23668/psycharchives.15528).
