# Supplementary material for: Interpersonal emotion regulation and symptom dimensions of psychosis proneness in young adults
Source: Schizophrenia (Heidelb). 2024 Nov 1;10(1):100. doi: 10.1038/s41537-024-00520-x (PMC11528099; doi:10.1038/s41537-024-00520-x)

Supplementary Material for:

Interpersonal Emotion Regulation and Dimensions of Psychosis Proneness in Young Adults

Marcel Riehle[1], Hannah Allmandinger[1] & Luise Pruessner[2]

[1]Universität Hamburg, Institute for Psychology

[2]Heidelberg University, Department of Psychology

– Supplementary Material –

This supplementary material contains supplementary text detailing methodological procedures, supplementary tables, and supplementary figures presenting additional results in more depth than the main manuscript.

Contents

Table S1. Demographic data and descriptive statistics. 3

Table S2. Pearson correlation coefficients matrix for age, gender, minority status, and self-reported questionnaires (CAPE, IERQ, ERQ). 4

Table S3. Full model results for the multiple regression testing for associations of IERQ-EP with CAPE symptom domains controlling for gender, minority status, and age. 5

Table S4. Full model results for the multiple regression testing for associations of IERQ-SM with CAPE symptom domains controlling for gender, minority status, and age. 5

Table S5. Full model results for the multiple regression testing for associations of IERQ-SO with CAPE symptom domains controlling for gender, minority status, and age. 6

Table S6. Full model results for the multiple regression testing for associations of IERQ-PT with CAPE symptom domains controlling for gender, minority status, and age. 6

Table S7. Full model results for the multiple regression testing for associations of ERQ-ES with CAPE symptom domains controlling for gender, minority status, and age. 7

Table S8. Full model results for the multiple regression testing for associations of ERQ-CR with CAPE symptom domains controlling for gender, minority status, and age. 7

Multiple imputation method 8

Figure S1. Density plot illustrating the distribution of the imputed vs. the observed values for CAPE – positive symptoms. 9

Figure S2. Density plot illustrating the distribution of the imputed vs. the observed values for CAPE – negative symptoms. 9

Figure S3. Density plot illustrating the distribution of the imputed vs. the observed values for CAPE – depression. 10

Figure S4. Density plot illustrating the distribution of the imputed vs. the observed values for IERQ – enhancement of positive affect. 10

Figure S5. Density plot illustrating the distribution of the imputed vs. the observed values for IERQ – social modeling 11

Figure S6. Density plot illustrating the distribution of the imputed vs. the observed values for IERQ – soothing. 11

Figure S7. Density plot illustrating the distribution of the imputed vs. the observed values for IERQ – perspective taking. 12

Figure S8. Density plot illustrating the distribution of the imputed vs. the observed values for ERQ – emotional suppression. 12

Figure S9. Density plot illustrating the distribution of the imputed vs. the observed values for ERQ – cognitive reappraisal 13

Table S1. Demographic data and descriptive statistics.

	N	%	*M* ± *SD*
**Age**	420		22.91 ± 3.08
**Gender (%)**			
Female	335	79.76	
Male	80	19.05	
Diverse	5	1.19	
**Education (%)**			
No school leaving certificate	2	0.48	
Certificate of Secondary Education	2	0.48	
General Certificate of Secondary Education	10	2.38	
Vocational diploma	26	6.19	
General qualification for university entrance	296	70.48	
Vocational training/Dual study program	20	4.76	
University degree	64	15.24	
**Cohabitants in household**	420		1.75 ± 1.68
**Relationship status (%)**			
Single	197	46.90	
In a relationship, not living together	134	31.90	
In a relationship, living together	77	18.33	
Married, living together	12	2.86	
**Minority status (%)**			
Yes	87	20.71	
No	333	79.29	
**CAPE**			
Positive	420		0.48 ± 0.31
Negative	420		1.06 ± 0.47
Depression	420		1.11 ± 0.51
**IERQ**			
Enhancement of positive affect	420		3.93 ± 0.71
Social modeling	420		3.37 ± 0.80
Soothing	420		3.17 ± 0.89
Perspective taking	420		2.23 ± 0.80
**ERQ**			
Emotional suppression	420		3.46 ± 1.20
Cognitive reappraisal	420		4.25 ± 1.11

Note. CAPE pos/neg/dep = Community Assessment of Psychic Experiences – attenuated positive/negative symptoms and depression. IERQ-EP/-EM/-SO-PT = Interpersonal Emotion Regulation Questionnaire – enhancement of positive affect/social modeling/soothing/perspective taking. ERQ-ES/-CR = Emotion Regulation Questionnaire – emotional suppression/cognitive reappraisal. Questionnaire M and SD are based on 30 multiply imputed data sets.

Table S2. Pearson correlation coefficients matrix for age, gender, minority status, and self-reported questionnaires (CAPE, IERQ, ERQ).

	Gender	Minority	CAPE pos	CAPE neg	CAPE dep	IERQ-EP	IERQ-SM	IERQ -SO	IERQ-PT	ERQ-ES	ERQ-CR
Age	0.09	0.01	-0.08	-0.03	-0.03	-0.07	0.04	0.07	0.06	-0.07	0.07
Gender		0.05	0.04	-0.02	**-0.11**	**-0.13**	**-0.15**	**-0.12**	0.03	0.06	0.03
Minority			**0.14**	0.08	**0.14**	-0.03	-0.01	0.04	-0.09	0.06	0.01
CAPE pos				**0.41**	**0.34**	-0.04	0.03	-0.02	**0.21**	**0.14**	-0.03
CAPE neg					**0.70**	**-0.24**	**-0.13**	**-0.14**	**-0.12**	**0.38**	**-0.19**
CAPE dep						-0.06	-0.06	0.03	**-0.16**	**0.26**	**-0.23**
IERQ-EP							**0.37**	**0.36**	**0.18**	**-0.15**	**0.22**
IERQ-SM								**0.44**	**0.41**	**-0.16**	**0.24**
IERQ-SO									**0.25**	**-0.43**	**0.14**
IERQ-PT										-0.07	**0.25**
ERQ-ES											0.03

Note. N = 420. CAPE pos/neg/dep = Community Assessment of Psychic Experiences – attenuated positive/negative symptoms and depression. IERQ-EP/-EM/-SO-PT = Interpersonal Emotion Regulation Questionnaire – enhancement of positive affect/social modeling/soothing/perspective taking. ERQ-ES/-CR = Emotion Regulation Questionnaire – emotional suppression/cognitive reappraisal. Gender was scored 0 = female, 1 = male. Diverse participants were coded as male and female in 50% of the 30 imputation data sets each. Correlation coefficients were calculated based on the 30 multiply imputed data sets and their *p*-values were determined based on Rubin's rules. The significance threshold was determined using the Benjamini-Hochberg correction with a false discovery rate of 5%. Significant correlations after correction ($p \leq 0.024$) are marked in bold font.

Table S3. Full model results for the multiple regression testing for associations of IERQ-EP with CAPE symptom domains controlling for gender, minority status, and age.

Fixed effects	***β***	SE	*t*	df	*p*	RIV	λ	FMI
Intercept	0.07	0.06	1.19	393.64	.234	0.03	0.03	0.04
Gender	-0.28	0.12	-2.29	337.17	.022	0.10	0.09	0.10
Minority	-0.06	0.12	-0.46	367.08	.646	0.07	0.06	0.07
Age	-0.06	0.05	-1.17	362.03	.243	0.07	0.07	0.07
**CAPE dep**	**0.19**	**0.07**	**2.66**	**327.69**	**.008**	0.11	0.10	0.11
**CAPE neg**	**-0.40**	**0.07**	**-5.59**	**343.89**	**<.001**	0.09	0.08	0.09
CAPE pos	0.06	0.05	1.19	356.09	.233	0.08	0.07	0.08
**Model**	**Estimate**	**95% CI Lower**	**95% CI Upper**	**FMI**				
R^2	0.10	0.05	0.16	0.08				

Note. N = 420. CAPE pos/neg/dep = Community Assessment of Psychic Experiences – attenuated positive/negative symptoms and depression. IERQ-EP = Interpersonal Emotion Regulation Questionnaire – enhancement of positive affect. RIV = Relative increase in variance. λ =Proportion of total variance due to the missing data. FMI = Fraction of missing information. The degrees of freedom are calculated based on Rubin's rules. P-values were adjusted using the Benjamini-Hochberg correction with a false discovery rate (FDR) of 5%. Significant fixed effects after correction (p ≤ 0.021) are marked in bold font.

Table S4. Full model results for the multiple regression testing for associations of IERQ-SM with CAPE symptom domains controlling for gender, minority status, and age.

Fixed effects	***β***	SE	*t*	df	*p*	RIV	λ	FMI
Intercept	0.09	0.06	1.44	394.60	.152	0.03	0.03	0.03
**Gender**	**-0.40**	**0.13**	**-3.19**	**346.74**	**.002**	0.09	0.09	0.09
Minority	-0.02	0.13	-0.14	349.08	.890	0.09	0.08	0.09
Age	0.06	0.05	1.17	359.32	.242	0.07	0.07	0.07
CAPE dep	0.02	0.07	0.31	334.96	.757	0.10	0.09	0.10
**CAPE neg**	**-0.19**	**0.07**	**-2.62**	**350.71**	**.009**	0.08	0.08	0.08
CAPE pos	0.11	0.06	2.02	355.06	.044	0.08	0.08	0.08
**Model**	**Estimate**	**95 % CI Lower**	**95% CI Upper**	**FMI**				
R^2	0.05	0.02	0.11	0.09				

Note. N = 420. CAPE pos/neg/dep = Community Assessment of Psychic Experiences – attenuated positive/negative symptoms and depression. IERQ-SM = Interpersonal Emotion Regulation Questionnaire – social modeling. RIV = Relative increase in variance. λ =Proportion of total variance due to the missing data. FMI = Fraction of missing information. The degrees of freedom are calculated based on Rubin's rules. P-values were adjusted using the Benjamini-Hochberg (BH) correction with a false discovery rate (FDR) of 5%. Significant fixed effects after BH correction (p ≤ 0.021) are marked in bold font.

Table S5. Full model results for the multiple regression testing for associations of IERQ-SO with CAPE symptom domains controlling for gender, minority status, and age.

Fixed effects	*β*	SE	*t*	df	*p*	RIV	λ	FMI
Intercept	0.04	0.06	0.69	395.54	.492	0.03	0.03	0.03
Gender	-0.29	0.12	-2.24	357.45	.026	0.07	0.07	0.08
Minority	0.08	0.12	0.63	354.64	.531	0.07	0.07	0.08
Age	0.08	0.05	1.69	346.44	.096	0.08	0.08	0.09
**CAPE dep**	**0.23**	**0.07**	**3.22**	**351.19**	**.001**	0.08	0.08	0.08
**CAPE neg**	**-0.32**	**0.07**	**-4.51**	**372.55**	**<.001**	0.06	0.06	0.06
CAPE pos	0.04	0.05	072	350.56	.472	0.08	0.08	0.08
**Model**	**Estimate**	**95% CI Lower**	**95% CI Upper**	**FMI**				
R^2	0.07	0.03	0.13	0.07				

Note. N = 420. CAPE pos/neg/dep = Community Assessment of Psychic Experiences – attenuated positive/negative symptoms and depression. IERQ-SO = Interpersonal Emotion Regulation Questionnaire – soothing. RIV = Relative increase in variance. λ =Proportion of total variance due to the missing data. FMI = Fraction of missing information. The degrees of freedom are calculated based on Rubin's rules. P-values were adjusted using the Benjamini-Hochberg (BH) correction with a false discovery rate (FDR) of 5%. Significant fixed effects after BH correction ($p \leq 0.021$) are marked in bold font.

Table S6. Full model results for the multiple regression testing for associations of IERQ-PT with CAPE symptom domains controlling for gender, minority status, and age.

Fixed effects	*β*	SE	*t*	df	*p*	RIV	λ	FMI
Intercept	0.06	0.06	1.00	393.26	.317	0.03	0.03	0.04
Gender	-0.02	0.12	-0.15	339.58	.881	0.10	0.09	0.09
Minority	-0.26	0.12	-2.14	339.78	.033	0.10	0.09	0.09
Age	0.09	0.05	1.80	370.96	.073	0.06	0.06	0.06
**CAPE dep**	**-0.16**	**0.07**	**-2.31**	**332.51**	**.021**	0.11	0.10	0.10
CAPE neg	-0.14	0.07	-1.96	366.36	.050	0.07	0.06	0.07
**CAPE pos**	**0.34**	**0.05**	**6.32**	**323.92**	**<.001**	0.12	0.10	0.11
**Model**	**Estimate**	**95% CI Lower**	**95% CI Upper**	**FMI**				
R^2	0.13	0.07	0.20	0.09				

Note. N = 420. CAPE pos/neg/dep = Community Assessment of Psychic Experiences – attenuated positive/negative symptoms and depression. IERQ-PT = Interpersonal Emotion Regulation Questionnaire – perspective taking. RIV = Relative increase in variance. λ =Proportion of total variance due to the missing data. FMI = Fraction of missing information. The degrees of freedom are calculated based on Rubin's rules. P-values were adjusted using the Benjamini-Hochberg (BH) correction with a false discovery rate (FDR) of 5%. Significant fixed effects after BH correction ($p \leq 0.021$) are marked in bold font.

Table S7. Full model results for the multiple regression testing for associations of ERQ-ES with CAPE symptom domains controlling for gender, minority status, and age.

Fixed effects	*β*	SE	*t*	df	*p*	RIV	λ	FMI
Intercept	-0.05	0.06	-0.91	403.00	.364	0.02	0.02	0.02
Gender	0.17	0.12	1.42	357.31	.155	0.08	0.07	0.08
Minority	0.08	0.12	0.68	368.59	.498	0.06	0.06	0.07
Age	-0.07	0.05	-1.45	341.77	.148	0.10	0.09	0.09
CAPE dep	-0.01	0.07	-0.11	335.20	.911	0.10	0.09	0.10
**CAPE neg**	**0.39**	**0.07**	**5.67**	**351.61**	**<.001**	0.08	0.08	0.08
CAPE pos	-0.03	0.05	-0.55	316.53	.585	0.12	0.11	0.12
**Model**	**Estimate**	**95% CI Lower**	**95% CI Upper**	**FMI**				
R^2	0.15	0.09	0.22	0.09				

Note. N = 420. CAPE pos/neg/dep = Community Assessment of Psychic Experiences – attenuated positive/negative symptoms and depression. ERQ-ES = Emotion Regulation Questionnaire – emotional suppression. RIV = Relative increase in variance. **λ** =Proportion of total variance due to the missing data. FMI = Fraction of missing information. The degrees of freedom are calculated based on Rubin's rules. P-values were adjusted using the Benjamini-Hochberg (BH) correction with a false discovery rate (FDR) of 5%. Significant fixed effects after BH correction ($p \leq 0.021$) are marked in bold font.

Table S8. Full model results for the multiple regression testing for associations of ERQ-CR with CAPE symptom domains controlling for gender, minority status, and age.

Fixed effects	*β*	SE	*t*	df	*p*	RIV	λ	FMI
Intercept	-0.02	0.06	-0.27	396.53	.788	0.03	0.03	0.03
Gender	-0.01	0.13	-0.12	345.63	.906	0.09	0.08	0.09
Minority	0.09	0.12	0.74	353.01	.464	0.08	0.08	0.08
Age	0.07	0.05	1.31	343.83	.189	0.09	0.09	0.09
**CAPE dep**	**-0.21**	**0.07**	**-2.92**	**351.53**	**.004**	0.08	0.08	0.08
CAPE neg	-0.08	0.07	-1.08	321.89	.279	0.11	0.11	0.11
CAPE pos	0.08	0.05	1.39	343.95	.164	0.09	0.08	0.09
**Model**	**Estimate**	**95% CI Lower**	**95% CI Upper**	**FMI**				
R^2	0.07	0.03	0.12	0.08				

Note. N = 420. CAPE pos/neg/dep = Community Assessment of Psychic Experiences – attenuated positive/negative symptoms and depression. ERQ-CR = Emotion Regulation Questionnaire – cognitive reappraisal. RIV = Relative increase in variance. **λ** =Proportion of total variance due to the missing data. FMI = Fraction of missing information. The degrees of freedom are calculated based on Rubin's rules. P-values were adjusted using the Benjamini-Hochberg (BH) correction with a false discovery rate (FDR) of 5%. Significant fixed effects after BH correction ($p \leq 0.021$) are marked in bold font.

Multiple imputation method

We imputed missing data using Multivariate Imputation by Chained Equations (MICE) and realized this via the *mice* package in R. The imputation model included the following variables: gender, age, minority status, CAPE positive symptoms, CAPE negative symptoms, CAPE depression, IERQ-EP, IERQ-SM, IERQ-SO, IERQ-PT, ERQ-ES, ERQ-CR, and an assessment of social distancing due to the Corona pandemic (SocDis) that was not further analyzed in the present study.

We drew 30 imputed data sets using 30 maximum iterations per imputation and predictive mean matching as described in Woods et al. (Woods et al., 2024. Best practices for addressing missing data through multiple imputation. *Infant and Child Development*, *33*(1), e2407, https://doi.org/10.1002/icd.2407).

Data were missing in 29 cases (6.9%) and, additionally, the answering pattern of 10 participants (2.4%) was determined unreliable. This was based either on failing more than two of the four attention checks (n = 8 [1.9%]) or, in case one or two attention checks were failed (n = 12 [2.9%]), by inspecting the answering pattern visually (particularly for long string answering). For participants whose data were determined unreliable, the demographic data were retained and all questionnaire responses were set to missing. Accordingly, overall, data for 41 participants (9.8% were imputed). Because participants were included in the analyses only if they had provided at least the demographic data, there were no missings for gender, age, and minority status. For the imputed variables, the rates of missing data were as follows: IERQ-EP ($n_{missing}$ = 30 [4.8%]), IERQ-SM ($n_{missing}$ = 30 [4.8%]), IERQ-SO ($n_{missing}$ = 30 [4.8%]), IERQ-PT ($n_{missing}$ = 30 [4.8%]), ERQ-ES ($n_{missing}$ = 35 [6.1%]), ERQ-CR ($n_{missing}$ = 35 [6.1%]), CAPE positive symptoms ($n_{missing}$ = 38 [6.9%]), CAPE negative symptoms ($n_{missing}$ = 38 [6.9%]), CAPE depression ($n_{missing}$ = 38 [6.9%]), SocDis ($n_{missing}$ = 41 [7.6%]).

Figures S1-S9 show the distribution of the imputed values plotted against the distribution of the observed (i.e., non-missing) values. As shown in these figures, the imputation produced reasonably distributed values.

Figure S1. Density plot illustrating the distribution of the imputed vs. the observed values for CAPE – positive symptoms.

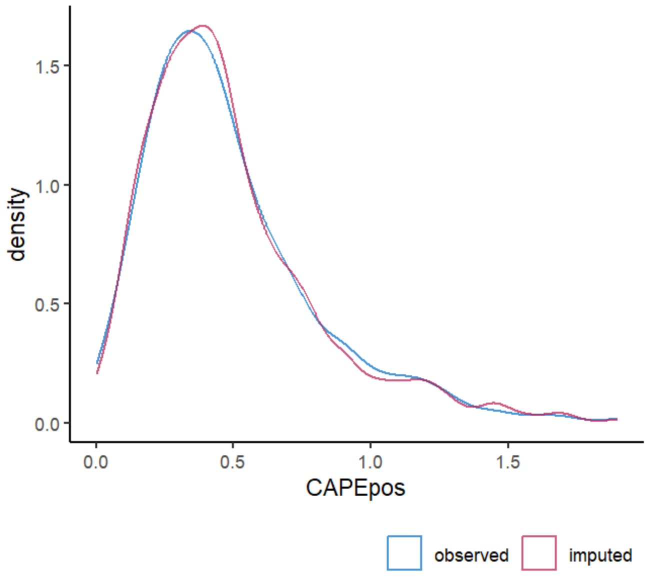

Figure S2. Density plot illustrating the distribution of the imputed vs. the observed values for CAPE – negative symptoms.

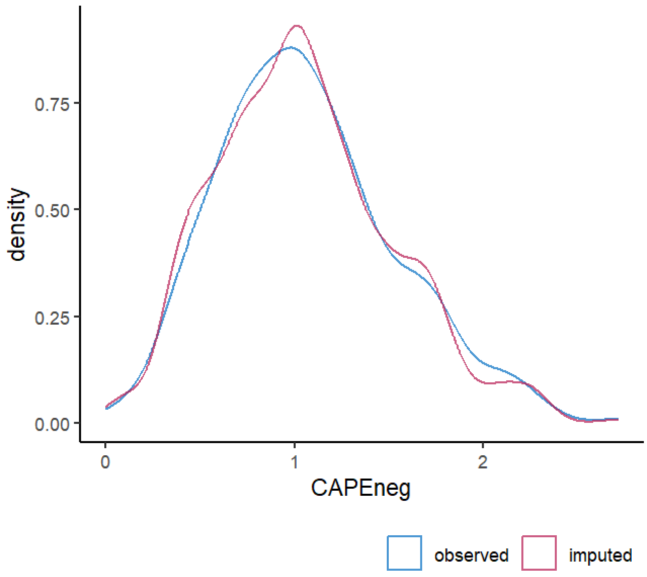

Figure S3. Density plot illustrating the distribution of the imputed vs. the observed values for CAPE – depression.

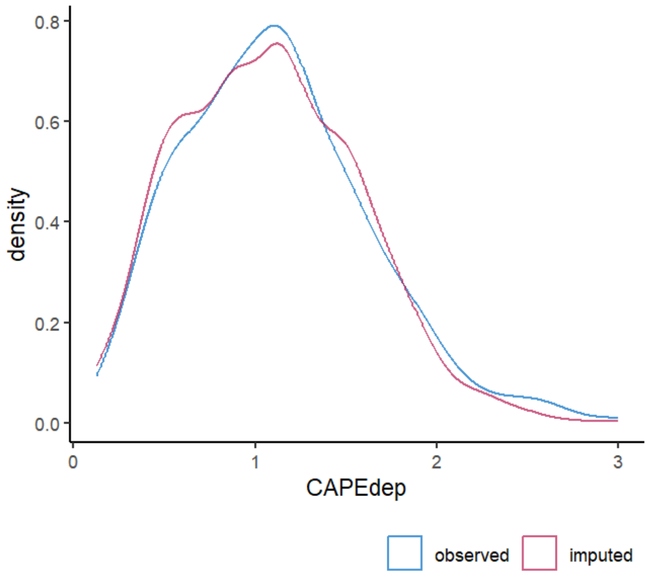

Figure S4. Density plot illustrating the distribution of the imputed vs. the observed values for IERQ – enhancement of positive affect.

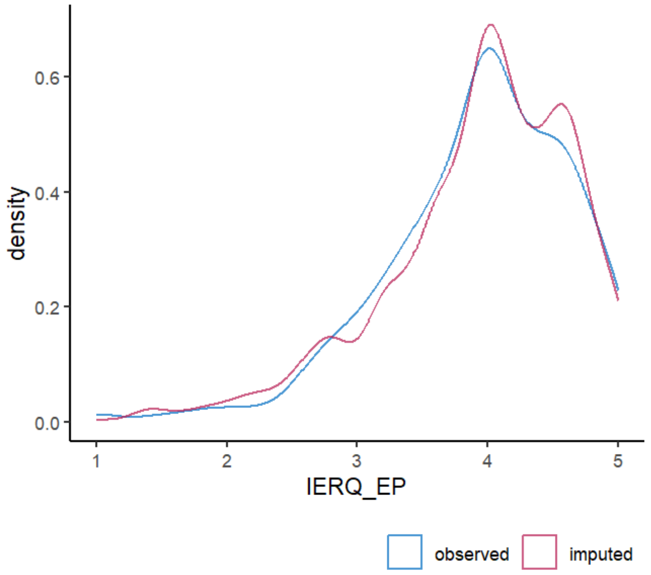

Figure S5. Density plot illustrating the distribution of the imputed vs. the observed values for IERQ – social modeling.

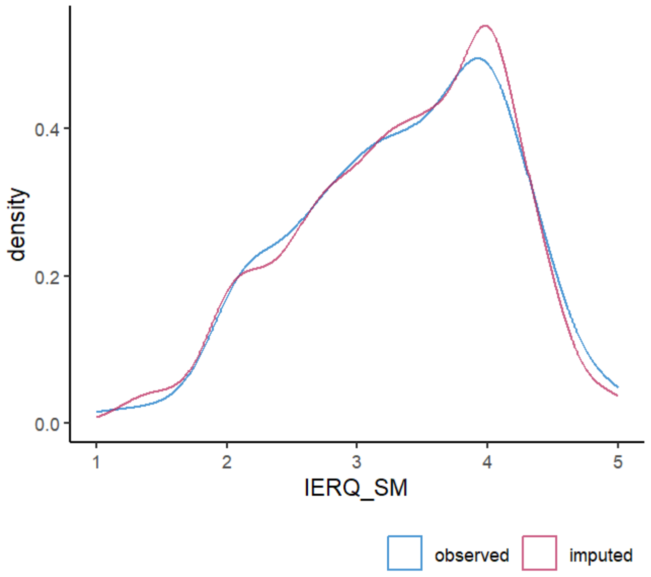

Figure S6. Density plot illustrating the distribution of the imputed vs. the observed values for IERQ – soothing.

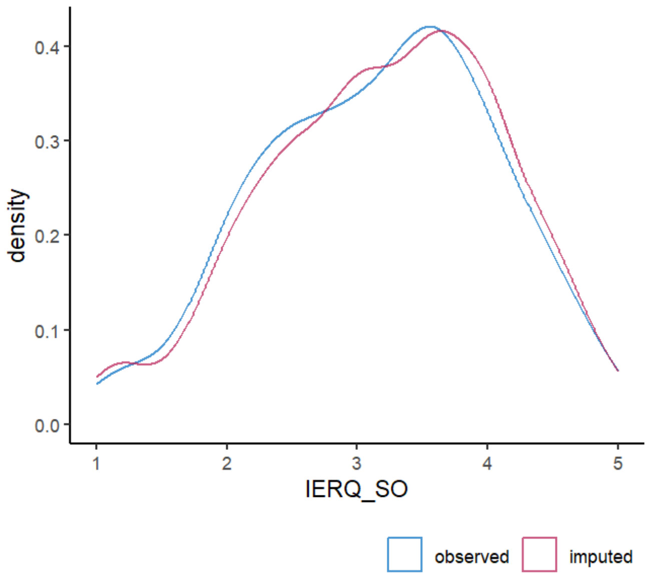

Figure S7. Density plot illustrating the distribution of the imputed vs. the observed values for IERQ – perspective taking.

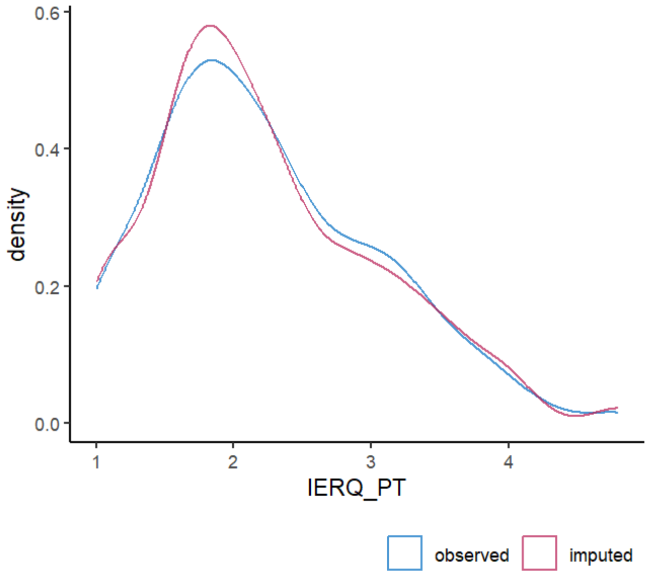

Figure S8. Density plot illustrating the distribution of the imputed vs. the observed values for ERQ – emotional suppression.

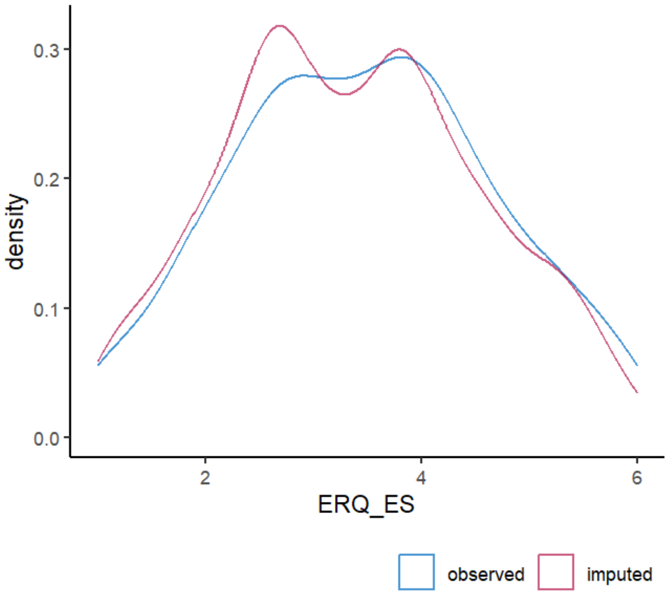

Figure S9. Density plot illustrating the distribution of the imputed vs. the observed values for ERQ – cognitive reappraisal.

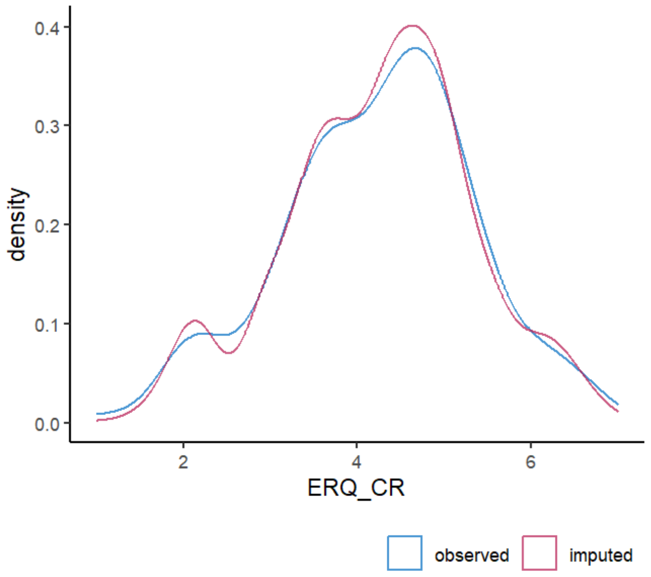

Supplement: Supplementary file 1 — Supplementary material [file 41537_2024_520_MOESM1_ESM.pdf]
